# Inter-phylum negative interactions affect soil bacterial community dynamics and functions during soybean development under long-term nitrogen fertilization

**DOI:** 10.1007/s44154-021-00015-0

**Published:** 2021-11-26

**Authors:** Chunfang Zhang, Shuo Jiao, Duntao Shu, Gehong Wei

**Affiliations:** State Key Laboratory of Crop Stress Biology for Arid Areas, Shaanxi Key Laboratory of Agricultural and Environmental Microbiology, College of Life Sciences, Northwest A&F University, Yangling, 712100 Shaanxi China

**Keywords:** Nitrogen management, Soybean development, Community spatiotemporal variations, Bacterial interactions, Functional groups, Nitrogen-cycling genes

## Abstract

Understanding interspecies interactions is essential to predict the response of microbial communities to exogenous perturbation. Herein, rhizospheric and bulk soils were collected from five developmental stages of soybean, which grew in soils receiving 16-year nitrogen inputs. Bacterial communities and functional profiles were examined using high-throughput sequencing and quantitative PCR, respectively. The objective of this study was to identify the key bacterial interactions that influenced community dynamics and functions. We found that the stages of soybean development outcompeted nitrogen fertilization management in shaping bacterial community structure, while fertilization treatments significantly shaped the abundance distribution of nitrogen functional genes. Temporal variations in bacterial abundances increased in bulk soils, especially at the stage of soybean branching, which helps to infer underlying negative interspecies interactions. Members of *Cyanobacteria* and *Actinobacteria* actively engaged in inter-phylum negative interactions in bulk soils and soybean rhizosphere, respectively. Furthermore, the negative interactions between nitrogen-fixing functional groups and the reduction of *nifH* gene abundance were coupled during soybean development, which may help to explain the linkages between population dynamics and functions. Overall, these findings highlight the importance of inter-phylum negative interactions in shaping the correlation patterns of bacterial communities and in determining soil functional potential.

**Supplementary Information:**

The online version contains supplementary material available at 10.1007/s44154-021-00015-0.

## Introduction

The soil microbiome plays an integral role in nutrient cycling and constitutes an important resource for sustainable agricultural production (van der Heijden et al. 2008). However, long-term chemical fertilizer application threatens soil biodiversity and agroecological services (Zhong et al. 2010). In contrast, livestock manure addition contributes to the restoration of soil bacterial diversity (Sun et al. 2015), and increases soil enzyme activity (Li et al. 2015). Long-term fertilization changes microbial composition and function, which could be further altered during soybean development. The rapid growth of soybean can not only improve the nutrient uptake by soybean roots, but also promote the secretion of roots (Leggett and Frere 1971; Tanaka et al. 2008). The root exudates of legume plants can serve as the energy sources of some soil microbes (Sugiyama and Yazaki 2012). The soybean rhizosphere selects a subset of bacterial communities from the bulk soil based on some specific functions related to nitrogen, phosphorus, potassium, and iron metabolism, which is beneficial to the growth and nutrition of soybean (Mendes et al. 2014). Moreover, the soybean-rhizobium symbiosis enhances soil fertility due to biological nitrogen fixation (Liebman et al. 2012). Soybean cultivation as green manure offers a promising approach to ameliorate the negative impact on soil nutrients caused by the long-term application of chemical nitrogen fertilizer. Therefore, exploring how soil bacterial communities and nitrogen functional traits respond to soybean cultivation after long-term nitrogen fertilization could help to guide agricultural management.

Both exogenous and endogenous factors shape microbial communities and their functions (Konopka et al. 2015; Zhang et al. 2021). On the one hand, agricultural management and plant selection interactively affect soil microbial communities and nitrogen cycling (Schmidt et al. 2019). On the other hand, it is widely recognized that microbial interactions play important roles in shaping community dynamics and functioning (Asiloglu et al. 2021; Konopka et al. 2015). A common environmental biotechnology is to stabilize microbial interactions by controlling microbial inoculums and environmental conditions in order to optimize metabolic processes (Perez-Garcia et al. 2016). Therefore, the control of biological processes requires an understanding of microbial interactions (Zengler and Palsson 2012). Although the effects of plant and environmental factors on microbial communities have been well studied (Brown et al. 2020; Mendes et al. 2014), the consequences of bacterial interactions for communities and functions have not been fully understood.

Microorganisms exist in complex ecological webs. Microbial interactions within these ecological webs can have a positive impact, a negative impact or no impact on the species involved (Faust and Raes 2012). Co-occurrence model analysis indicates that interactions among root-associated fungal communities are predominantly positive (Abrego et al. 2020). Some studies have found that organic fertilizer amendment increases the ratio of bacteria to fungi, which is underpinned by the change of bacteria (Tao et al. 2015; Ye et al. 2021). Long-term manure application alters the composition of keystone taxa, and increases microbial network complexity (Ye et al. 2021). A previous study found that organic fertilizer reduced the population of pathogens, whilst antagonistic microbial groups negatively correlated with the pathogen populations (Tao et al. 2015). Furthermore, bacterial inoculants suppress pathogens, while increase the relative abundances of beneficial bacteria (Li et al. 2021; Zhao et al. 2021). These findings highlight that negative interactions between soil microbes can lead to microbial abundance variations. Hence, we hypothesize that bacterial negative interactions play important roles in shaping dynamic variations in community composition under the legacy effects of long-term fertilization.

It is challenging to measure interspecies interactions in the whole microbial community through direct observation. Since interaction states might reach large numbers with only a few highly connected taxa, the key is to find the key interactions that have percolating effects at the community level (Grosskopf and Soyer 2014). Microbial networks can be used to predict interactions between species and to identify keystone taxa (Agler et al. 2016; Faust and Raes 2012). A previous study found that keystone taxa could explain community turnover better than all taxa combined (Herren and McMahon 2018), suggesting that they could be harnessed to predict shifts in microbial community composition (Banerjee et al. 2018). Exploring the co-occurrence patterns of keystone taxa would help to identify the key interactions in microbial communities (Huang et al. 2016; Liu et al. 2020).

Microbial community composition could affect ecosystem functioning. However, microbial communities similar in taxonomic composition could be dissimilar in functional potentials (Strickland et al. 2009). A previous study suggested that only a subset of microbes were vital for the mediation of biogeochemical cycles (Zhao et al. 2014). Microbial taxonomic composition and functional structure tend to be shaped by different processes (Louca et al. 2018). A global biogeographical study found that soil properties largely explained the variations in nitrogen-cycling traits, while weakly explaining the taxonomic composition of the corresponding functional groups (Nelson et al. 2016). Some studies observed that the relative abundances of specific bacterial phyla were useful parameters for predicting differences in soil functions in agricultural ecosystems (Nazaries et al. 2021; Zhong et al. 2020). The correlation between microbial taxonomic and functional composition is becoming the central question in microbial ecology. Understanding their associations can help to make decisions on how to regulate biological processes through the management of soil microbiota.

Identifying the mechanisms and consequences of microbial interactions will provide useful principles for developing predictive understandings of community dynamics and for the design of robust synthetic communities (Konopka et al. 2015). Nevertheless, how bacterial interaction patterns influence the soil microbiome remains unexplored during soybean development under long-term nitrogen addition. Therefore, the objectives of this study were (1) to reveal the spatiotemporal variation patterns of the overall bacterial community and nitrogen-cycling traits, (2) to predict the key interactions between keystone taxa and to verify their percolating effects at the overall community level, and (3) to explore to what extent bacterial interactions influence the nitrogen-cycling processes.

## Results

### Spatiotemporal variations of bacterial community and nitrogen functional genes

Bacterial community differences were mainly driven by stage, and secondarily, by fertilization treatment. Soil compartment had a slight driving effect on the changes in community structure (Table 1). In view of the striking differences in bacterial community structure across different stages, the temporal effect was further analyzed using PCoA and PERMANOVA (Supplementary Fig. S2). Results showed that bacterial community structure became more similar between CK, N1, and N2, as well as between O1 and O2 over time (Supplementary Table S2). However, bacterial community structure consistently differentiated between organic and mineral fertilization treatments. Moreover, the difference in community structure increased between bulk and rhizosphere samples as the development of soybean progressed (Supplementary Fig. S2).

**Table 1:** Analysis of similarities (ANOSIM) and permutational multivariate analysis of variance (PERMANOVA)

Category^a^	Group	ANOSIM		PERMANOVA	
		***R***	***P***	***R***^**2**^	***P***
Bacteria	Fertilization	0.300	< 0.001	0.118	< 0.001
	Stage	0.565	< 0.001	0.205	< 0.001
	Compartment	0.157	< 0.001	0.038	< 0.001
Genes	Fertilization	0.383	< 0.001	0.442	< 0.001
	Stage	0.016	0.091	0.050	< 0.001
	Compartment	−0.003	0.534	0.003	0.005

^a^The analysis is based on bacterial communities and the abundances of nitrogen functional genes

**Fig. 1:**
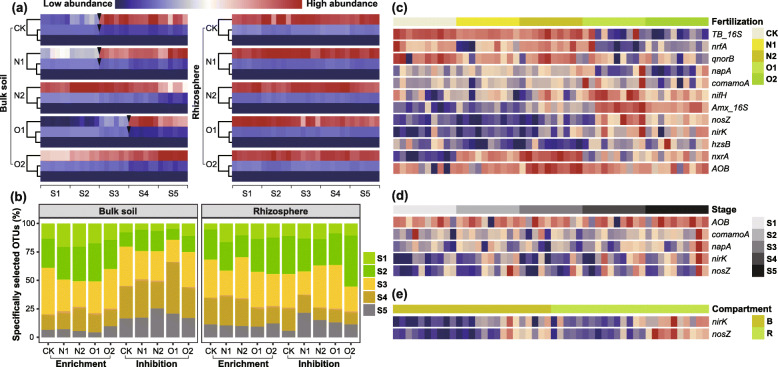
Spatiotemporal distribution of bacterial communities and nitrogen functional genes. **a** Abundance variation patterns of OTUs on time scales. Each stage includes six replicates. OTUs are aggregated with three kmeans clusters in each treatment. **b** Proportions of the OTUs that have the highest or lowest relative abundances across all stages. **c-e** Average abundances of significantly-differentiated functional genes between different sample groups. Each average gene abundance is calculated from three replicates for each sample. The heatmap legend of Fig. 1c-e is shown in Fig. 1a. *TB_16S*, 16S rRNA genes of total bacteria; *Amx_16S*, 16S rRNA genes of anammox bacteria; *AOB*, *AOB amoA*; *comamoA*, *comammox clade A*; CK, no fertilizer; N1, low urea input; N2, high urea input; O1, low sheep manure input; O2, high sheep manure input. B, bulk soil; R, rhizosphere; S1, S2, S3, S4, and S5 represent one, three, five, seven, and nine weeks post transplantation, respectively

The species that responded consistently to one treatment in six replicates could be clustered into three groups based on their abundance variation patterns (Fig. 1a, Supplementary Table S3). Operational taxonomic units (OTUs) within these three groups exhibited relatively uniform abundance distribution in rhizosphere samples across time scales. However, two groups of OTUs with high abundances showed opposite directions in abundance change across bulk samples at specific transition points (Fig. 1a), which suggested that some species were selected for while others were selected against at particular stages. In the rhizosphere, the periods when OTUs were specifically enriched or inhibited in large amounts were generally synchronous (Fig. 1b). In the bulk soil, around 60% of OTUs were specifically enriched at S2 and S3, and specifically inhibited at S3 and S4. Such specifically enriched OTUs were significantly more and less than specifically inhibited OTUs at S2 and S4, respectively (Fig. 1b, Supplementary Table S4). These results suggested that the asynchrony of enrichment and inhibition processes increased variation in bacterial community abundance.

The results of ANOSIM showed that the variation pattern of nitrogen functional genes was only determined by nitrogen fertilization management (Table 1). The driving effect of stages on variation in the abundance of functional genes could not be observed. Significance tests showed that all the analyzed genes significantly varied in abundance among different fertilization treatments. The genes involved in nitrogen fixation and anammox processes had higher abundances in O1 and O2 than in N1 and N2 treatments, with a fold change value of 2.6 for *nifH* gene and 2.4 for the 16S rRNA gene of anammox bacteria. However, the *nrfA* gene showed an opposite trend (Fig. 1c). In addition, five genes showed significant temporally-dependent abundance variation, including two in the nitrification process and three in the denitrification process. In particular, the abundances of the *AOB amoA* gene were significantly lower at S1 and S2 than at the later stages (Fig. 1d). Moreover, *nirK* and *nosZ* genes had significantly higher abundances in rhizosphere than in bulk samples (Fig. 1e).

### Interaction patterns of temporally specifically-selected taxa

**Fig. 2:**
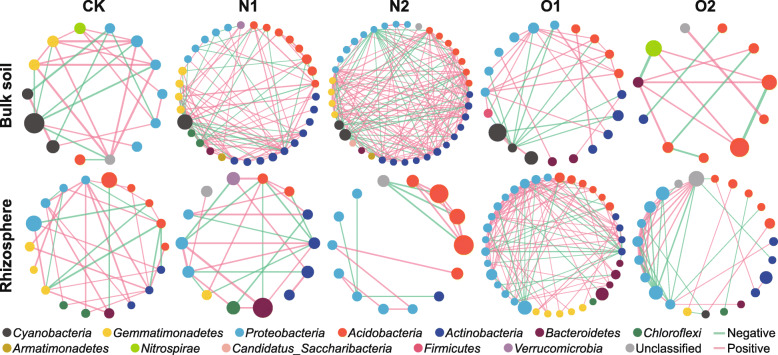
The sub-networks of the keystone taxa identified in the networks constructed from temporally specifically-selected sub-communities. Nodes represent keystone OTUs, and are colored by phyla. The node size is proportional to the relative abundance of OTUs in each compartment under different fertilization treatments. The edge width and color represent the size and direction of correlation coefficients, respectively. CK, no fertilizer; N1, low urea input; N2, high urea input; O1, low sheep manure input; O2, high sheep manure input

The observed networks of temporally specifically-selected sub-communities had higher average clustering coefficients, average path lengths, and modularity than the corresponding random networks (Supplementary Table S5). Keystone taxa were identified in the specifically selected sub-communities. The keystone members of *Cyanobacteria* negatively correlated with the keystone members of *Proteobacteria*, *Actinobacteria* and other keystone phyla in bulk samples, except for O2 treatment (Fig. 2). In contrast, the interactions among keystone *Cyanobacteria* were positive in bulk samples. However, the role of *Cyanobacteria* as keystone taxa was not detected in the rhizosphere samples. Most of these cyanobacterial taxa belonged to unclassified genera. With regard to the network interactions between keystone taxa in rhizosphere samples, *Burkholderia* sp. and *Beggiatoa* (members of *Proteobacteria*) were actively involved in the intra-phylum negative interactions in O1 and O2 treatments, respectively (Fig. 2).

**Fig. 3:**
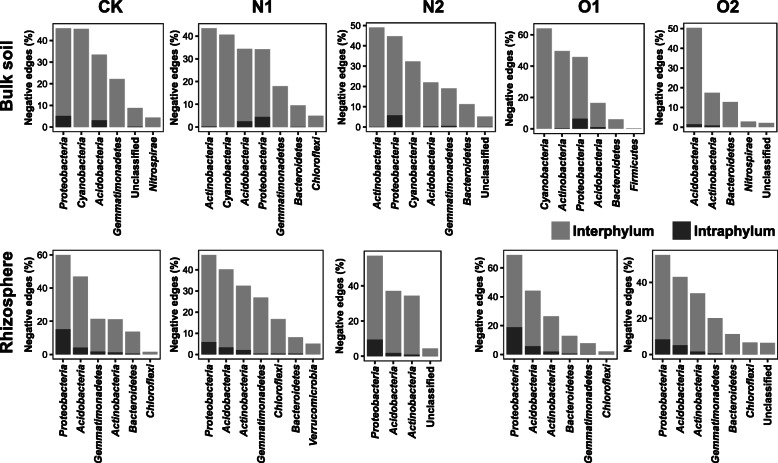
The proportions of negative correlations in the networks constructed from specifically selected sub-communities. Only negative correlations between the phyla containing keystone taxa are shown. CK, no fertilizer; N1, low urea input; N2, high urea input; O1, low sheep manure input; O2, high sheep manure input

Figure 3 showed that the interaction patterns of keystone taxa applied to the temporally specifically-selected sub-communities. For instance, *Cyanobacteria* actively engaged in the inter-phylum negative interactions among the specifically selected sub-communities in bulk samples, particularly under low organic nitrogen treatment. However, the proportions of negative interactions involved with *Cyanobacteria* decreased, while the contribution of *Actinobacteria* to negative interactions increased, under inorganic nitrogen treatments in bulk samples. In addition, *Proteobacteria*, *Acidobacteria*, and *Actinobacteria* tended to engage in inter- and intra-phylum negative interactions among the specifically selected sub-communities, especially in rhizosphere samples (Fig. 3).

### Inter-phylum associations at the overall community level

**Fig. 4:**
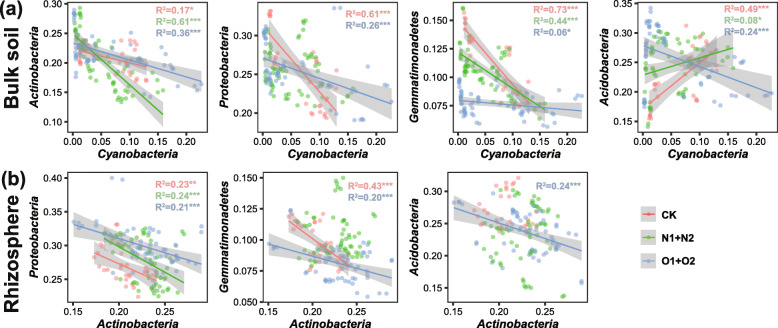
Pairwise associations between key phyla based on their relative abundances in **a** the bulk soil and **b** rhizosphere at the community level. Trend lines and goodness-of-fit indexes are added when relationships between variables are significant in linear models. ^*^P < 0.05; ^**^P < 0.01; ^***^P < 0.001

We further verified whether the negative interactions involved with keystone taxa contribute to the correlation patterns of the overall bacterial community. Generally, the pairwise relationships showed that the abundance correlations between *Cyanobacteria* and the other key phyla were stronger in bulk soils (Fig. 4a) than in rhizosphere (Supplementary Fig. S3a). Specifically, *Cyanobacteria* had negative correlations with *Actinobacteria*, *Proteobacteria*, and *Gemmatimonadetes* in bulk samples. However, *Cyanobacteria* negatively and positively correlated with *Acidobacteria* under organic and inorganic treatments, respectively, in bulk samples (Fig. 4a).

In addition to the stronger correlations in bulk samples, there were also some stronger abundance relationships in rhizosphere samples, and all the pairwise associations related to *Actinobacteria* showed higher goodness-of-fit indexes in rhizosphere (Fig. 4b) compared to the bulk soil (Supplementary Fig. S3b). Specifically, *Actinobacteria* strongly and negatively correlated with *Proteobacteria* and *Gemmatimonadetes* in almost all treatments across rhizosphere samples. Moreover, the negative linkages between *Actinobacteria* and *Acidobacteria* were strong only under organic nitrogen treatments in rhizosphere samples (Fig. 4b). Furthermore, redundancy analysis also demonstrated that *Cyanobacteria* and *Actinobacteria* negatively correlated with other key phyla in the bulk soil and rhizosphere, respectively (Supplementary Fig. S4).

### Contribution of functional groups to nitrogen cycling

*Cyanobacteria* greatly affected the abundances of nitrogen functional genes under the organic nitrogen treatments in the bulk soil based on redundancy analysis (Supplementary Fig. S4). In addition, the 16S rRNA gene of anammox bacteria, as well as *nosZ*, *AOB amoA*, *nifH*, and *nirK* genes showed positive correlations with *Cyanobacteria*, and *qnorB* and *nifH* genes were positively correlated with *Proteobacteria* in the bulk soil. While *Cyanobacteria* were not identified as keystone taxa in rhizosphere, they partly influenced the abundances of nitrogen functional genes in rhizospheric CK treatments. However, *Actinobacteria* greatly influenced the abundances of nitrogen functional genes together with *Proteobacteria* in rhizospheric organic treatments. *Actinobacteria* positively correlated with *AOB amoA* and *nxrA* genes, and *Proteobacteria* positively affected *nosZ* and *hzsB* genes in rhizosphere (Supplementary Fig. S4).

**Fig. 5:**
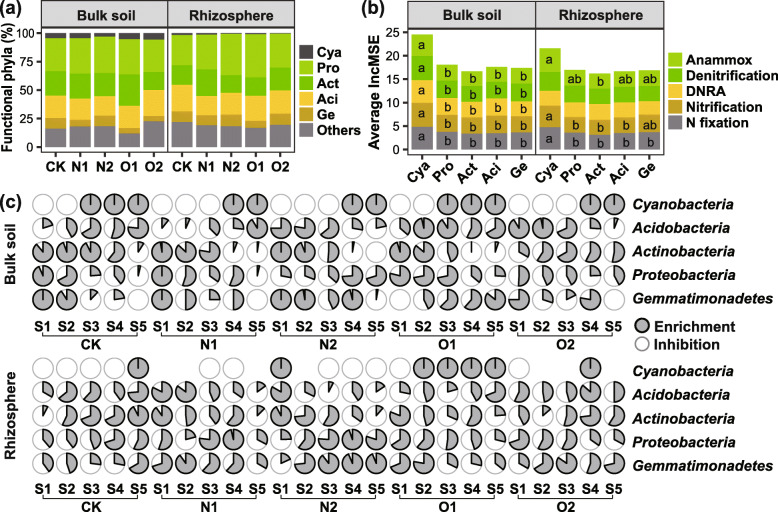
Functional phyla’s **a** composition, **b** relative importance to nitrogen cycling processes, and **c** selection specificity. The same means separation letters in each process within each compartment indicate that the values are not significantly different at *P* ≤ 0.05. Cya, *Cyanobacteria*; Pro, *Proteobacteria*; Act, *Actinobacteria*; Aci, *Acidobacteria*; Ge, *Gemmatimonadetes*; DNAR, dissimilatory nitrate reduction to ammonium; CK, no fertilizer; N1, low urea input; N2, high urea input; O1, low sheep manure input; O2, high sheep manure input. S1, S2, S3, S4, and S5 represent one, three, five, seven, and nine weeks post transplantation, respectively

The temporally specifically-selected OTUs that significantly predicted variations in the abundance of functional genes constituted functional groups. *Proteobacteria*, *Actinobacteria*, *Acidobacteria*, *Gemmatimonadetes*, and *Cyanobacteria* dominated the functional groups, except that *Cyanobacteria* only accounted for a small part of functional phyla in the rhizosphere (Fig. 5a). Some taxa within the phylum *Cyanobacteria* primarily contributed to variations in the abundance of the genes involved in multiple nitrogen-cycling processes, which was revealed by the highest IncMSE values (Fig. 5b).

Figure 5c showed that there was opposite selection specificity between functional groups at specific stages. For example, *Cyanobacteria* were inhibited at S1 and S2, and enriched at S4 and S5 in bulk samples. In contrast, most *Actinobacteria* were enriched at S1 and S2 in bulk samples, except for the O2 treatment. And *Actinobacteria* were inhibited at S4 and S5 under N1, N2, and O1 treatments in bulk samples (Fig. 5c). In rhizosphere, such opposite selection specificity between *Cyanobacteria* and *Actinobacteria* was detected at S2 and S3 under CK and O1 treatments, while this trend was not obvious under other treatments. Moreover, *Proteobacteria* and *Gemmatimonadetes* tented to be selected in the opposite direction to *Cyanobacteria* under CK in bulk samples. *Cyanobacteria* and *Acidobacteria* were oppositely selected under N2 and O2 treatments in bulk soils (Fig. 5c).

## Discussion

### Temporal variations of bacterial community shaped by rhizosphere effect

Root exudates can shape soil microbial communities during plant development (Chen et al. 2019). In this study, although long-term fertilization treatments maximized the differentiation between different systems, the temporal variation in bacterial community structure was more apparent than the variation caused by nitrogen fertilization treatments (Table 1). A recent study found that nitrogen limitation in the maize rhizosphere outweighed the effects of long-term fertilization on microbiome (Schmidt et al. 2019). Compared with maize, soybean was less dependent on soil nitrogen, since soybean nitrogen demand can be partially met (50–60%) by biological nitrogen fixation (Ortez et al. 2018). It is widely acknowledged that rhizodeposition is an important source of available carbon for microorganisms in the vicinity of growing roots (Butler et al. 2003). Root-released organic carbon, rather than soil organic carbon or soil active carbon, dramatically varied across wheat growth stages, and closely correlated with bacterial communities (Chen et al. 2019). Therefore, we supposed that the rhizosphere carbon flow related to soybean development affected bacterial community structure more considerably than nitrogen management strategies.

Competition for nutrients between two populations tends to result in the elimination of one population, especially when there is a single resource for competition and when there is no direct contact of these populations (Fredrickson and Stephanopoulos 1981). Herein, the more slight temporal variation in rhizospheric bacterial abundance is consistent with previous findings that microbial communities had less dynamic structure and greater ecological stability in rhizosphere than in the bulk soil (Costa et al. 2006; Thebault and Fontaine 2010). Carbon availability is much higher in rhizosphere than in bulk soils (Martin 2016). The rhizosphere can recruit specific bacteria with the ability to use diverse root-derived carbon substrates (Philippot et al. 2013). The continuous resource supply from root exudates potentially contributed to the reduced variation in the abundance of rhizospheric bacterial communities. Compared with the rhizosphere, the bulk soil obtains relatively limited resources and harbors microbes that are distributed more randomly (Fan et al. 2017). This would intensify bacterial competition for resources in bulk soils, and thereby caused dramatic changes in bacterial abundance.

Previous research has observed temporal shift in carbon utilization by rhizospheric microbial communities with the growth of soybean (Garland 1996). However, bacterial communities showed obvious abundance transitions in bulk soils at the branching stage (S2-S4), with most species enriched at S2 and then inhibited at S4. This can be partly explained by the fact that the rapid growth and nutrient uptake of soybean decrease substrate levels in the surrounding bulk soil at the branching stage (Leggett and Frere 1971; Tanaka et al. 2008), and further change bacterial substrate utilization and competition. Soybean root systems grow fast and display enhanced root exudation at the branching stage (Tanaka et al. 2008). Plant-derived carbon can enter the surrounding soil in the form of low molecular weight organic compounds (Hinsinger et al. 2005). The microbes that can assimilate the plant-derived carbon will be enriched in the bulk soil (Liu et al. 2016b). However, competition between the microbes that share common resources will eventually limit species abundance not to exceed the carrying capacity of the bulk soil, leading to the abundance decline of specific bacteria (Maslov and Sneppen 2017). These results suggested that the rhizosphere effect, especially at the stage of soybean branching, might affect bacterial abundance variation by enhancing bacterial trophic interaction in bulk soils.

### Endogenous and exogenous influences on bacterial community dynamics

A previous study found that the negative links within sub-networks and total networks were much greater in bulk soils than in wheat rhizosphere (Fan et al. 2018). We found a similar trend that the pairwise associations between *Cyanobacteria* and other phyla were stronger in bulk samples. This could be partly explained by the aforementioned increased competition for limited nutrients in bulk soils. However, *Proteobacteria*, *Gemmatimonadetes*, and *Acidobacteria* had stronger negative links with *Actinobacteria* in rhizosphere than in bulk samples. Given the contribution of *Actinobacteria* to the promotion of plant growth in rhizosphere (Singh et al. 2018), the interactions mediated by *Actinobacteria* could provide new insights into the mechanisms of *Actinobacteria* as bioinoculant for sustainable agriculture.

*Cyanobacteria* can accumulate extracellular polysaccharides (EPS) which can also be used by other soil microbes (Mager and Thomas 2011). We found that *Cyanobacteria* engaged in multiple inter-phylum negative interactions in both the specifically selected sub-communities and the overall communities across bulk soils. Compared with the rhizosphere, the bulk soil obtains relatively limited nutrients (Fan et al. 2017). The intensive bacterial competition for EPS in the bulk soil might lead to the negative correlations between *Cyanobacteria* and other bacteria. Previous studies have observed that nutrient variations oppositely impact the relative abundances of *Cyanobacteria* and *Actinobacteria* (Ghai et al. 2014; Ren et al. 2017). This inverse relationship between the members of *Cyanobacteria* and *Proteobacteria* was also observed in the coastal water of northern China (He et al. 2017). It has been reported that the occurrence and disappearance of cyanobacterial blooms linked to environmental heterogeneity induces shifts in microbial community composition (Xue et al. 2018). Herein, we found that correlation patterns in the bacterial community were characterized by negative interactions involved with *Cyanobacteria* in bulk soils.

We noted that *Actinobacteria* accounted for a larger proportion of negative links than *Cyanobacteria* in N1 and N2 treatments across bulk soils (Fig. 3), where their keystone taxa negatively and intensively interacted with each other (Fig. 2). This could be explained from two aspects. First, the cyanophycinase produced by *Actinobacteria* can enable them to directly degrade cyanophycin, which is an important storage polymer of carbon and nitrogen for several *Cyanobacteria* (Ghai et al. 2013). Second, *Actinobacteria* are sensitive to organic matter-enriched conditions, while *Cyanobacteria* positively respond to pulsed or sustained nutrient loads (Ghai et al. 2014). Previous studies identified several actinobacterial isolates as nitrate-dependent iron-oxidizers (Kanaparthi et al. 2013). In addition, *Actinobacteria* can grow efficiently in phosphorus-limited environment (Yao et al. 2016). As such, the increase in nitrate nitrogen and the reduction in available phosphorus concentrations (Supplementary Table S6) could explain the dominance of *Actinobacteria* under long-term urea addition treatments. Overall, both endogenous interactions and nitrogen management affected the interactive dynamics between *Actinobacteria* and *Cyanobacteria*.

Iron is a necessary nutrient for the metabolism of all living organisms (Cruz-Morales et al. 2017). Soybeans are prone to iron deficiency chlorosis, especially in alkaline soils (Rogers et al. 2009). When the *Bradyrhizobia* that serve as symbionts in soybean are defective in iron uptake and translocation, they elicit ineffective nodules, which do not contain leghemoglobin and lack the ability to fix nitrogen (Benson et al. 2005; Sankari and O'Brian 2016). Iron availability plays important roles in regulating the primary production and nitrogen fixation mediated by *Cyanobacteria* in marine ecosystems (Krupke et al. 2013). Hydroxamate siderophores (Desferrioxamines) are widely conserved in aquatic and soil-dwelling *Actinobacteria*, as well as in *Gammaproteobacteria* and *Alphaproteobacteria* (Cruz-Morales et al. 2017). Some actinobacterial species were capable of ammonium oxidation coupled to iron reduction (Feammox process) (Huang and Jaffé 2014). Considering the prominent roles of *Cyanobacteria*, *Actinobacteria*, and *Proteobacteria* in interspecies negative interactions, we suggest that the competition for iron via species-specific siderophores may influence bacterial interaction patterns and nitrogen-cycling processes in soybean production systems.

### Associations between bacterial communities and nitrogen functional genes

Previous research reported that microbial communities that were similar in taxonomic composition could have distinct functions, and thereby a subset of microbes might be the key players in mediating biogeochemical cycles (Fan et al. 2021; Zhao et al. 2014). In the present study, we found that bacterial community structure significantly changed over time. However, the abundances of nitrogen functional genes showed some degree of homogenization as the development of soybean. This temporal homogenization was previously reported in the microbial functional profiles of 1-year and 5-year soybean cultivation systems (Mendes et al. 2014). These results reinforced the idea that taxonomically distinct microorganisms could perform similar ecological functions. Apart from microbial functional redundancy, the reservoir role of rare species responding to environmental disturbance could also explain the abundance stability of functional genes (Mo et al. 2018).

As a previous study has highlighted, it is necessary to link the temporal turnover of microbial communities with their functional processes (Liang et al. 2015). Given the dilution effects of less active and inactive species, it is more feasible to explore the associations between community and functional genes by using the temporally specifically-selected sub-community (Yao et al. 2020). Herein, we explored the relative importance of temporally specifically-selected sub-communities to functional gene abundance using randomForest. If one species had higher IncMSE value in a certain nitrogen-cycling process, this species might directly perform functions, or indirectly interact with other functional taxa. We noticed that *Cyanobacteria* strongly predicted multiple nitrogen-cycling processes and actively engaged in inter-phylum negative interactions in bulk soils, which showed that *Cyanobacteria* tended to affect functional genes in an indirect way by interacting with other functional species.

The influence of microbial communities on microbial functions depends on the function measured (Griffiths et al. 2001). We found that the copy numbers of *nifH* gene and the associated IncMSE values showed significant negative correlations (Supplementary Fig. S5). For instance, the abundances of *nifH* gene were significantly lower in CK and N2 than in O1 treatments (Fig. 1c and Supplementary Fig. S6a). Meanwhile, they were predicted better by functional groups under CK and N2 than under O1 treatments. These results suggested that the abundance decline of nitrogen-fixing functional groups tended to decrease *nifH* gene abundances. Microbial abundance decline caused by environmental changes can decrease energy investment in nitrogen fixation and increase competition for nutrients (Camenzind et al. 2018). Both *Cyanobacteria* and rhizobial *Proteobacteria* are able to fix nitrogen (Louca et al. 2018; Warshan et al. 2017). Considering the strongly negative correlations between *Proteobacteria* and *Cyanobacteria* abundances under CK treatment in the bulk soil, their interplay might adversely affect the abundance of *nifH* gene. These results indicated that the negative interactions between nitrogen-fixing bacteria tended to be coupled with the reduction of *nifH* gene abundance. This observation could provide references for the better exploration of the correlation between microbial taxonomy and function.

Our findings demonstrate that *Cyanobacteria* and *Actinobacteria* have significant negative associations with other keystone phyla in bulk soils and the soybean rhizosphere, respectively. These results provide suitable targets for the future manipulation of the soybean microbiome. Although endogenous interactions and nitrogen management practices have partly explained the interactive dynamics between *Cyanobacteria* and *Actinobacteria*, further studies should focus on their interaction mechanisms and their roles in mediating nitrogen cycling and controlling crop production. Another important finding is that the negative interactions between nitrogen-fixing bacteria tends to adversely affect the abundance of *nifH* gene. This result provides a new perspective for revealing the linkages between the dynamics and functions of bacterial communities.

## Materials and methods

### Soil origin, soybean cultivation, and soil sampling

The soils used in the cultivation experiment were collected from the agricultural station of the Research Center on Agricultural Development Strategy in the Semi-arid area of China (108°04′E, 34°18′N). This region has a temperate continental monsoon climate, with average annual temperature and precipitation of 13 °C and 600 mm, respectively. The soil type is classified into Udic Haplustalf according to the USDA system (Soil-Survey-Staff 2010). Since 2002, a long-term fertilization experiment was conducted at this station. Winter wheat (*Triticum aestivum*) was annually fertilized with straw alone (CK; 4500 kg hm^− 2^) or in combination with 50% (low) or 100% (high) of nitrogen addition in the form of urea or sheep manure before sowing. The amounts of fertilizers applied were estimated according to their nitrogen contents to reach 120 kg hm^− 2^ nitrogen or 240 kg hm^− 2^ nitrogen applied to soil. The soil under CK treatment and the soils that received low urea (N1), low sheep manure (O1), high urea (N2), or high sheep manure (O2) were collected at the depth of 0–15 cm after harvest on July 16, 2018. Then the soils under the five different treatments were separately mixed and sieved through 2-mm meshes, and were used for soil property determination and the rhizobox experiment.

The soils used for physicochemical property analysis were transported in ice and stored at 4 °C. Soil pH was determined by a glass electrode with a dry soil to water ratio of 1:2.5. Air-dried soils were sieved through 1-mm meshes before measuring the concentrations of total carbon, total nitrogen, soil organic matter (Soman et al. 2017), available nitrogen including nitrate and ammonium nitrogen, available phosphorus, available potassium, and available iron based on published methods (Sun et al. 2015). The soils used for soybean (*Glycine max*, cv. Zhonghuang-13) cultivation were air-dried, and then adjusted to 60% of field capacity before the transplantation of soybean seedlings. Rhizobox (Supplementary Fig. S1) can separate rhizospheric soil from the bulk soil by 30-μm nylon net (Ling et al. 2012). Each fertilization treatment contained six replicate rhizoboxes with 400 g air-dried soil in each rhizobox.

Soybean seeds were surface sterilized with 2% sodium hypochlorite for 3 min, and then rinsed with sterile water six times before germination in the dark on 1% agar plates at 28 °C. Two germinated soybeans were transplanted into the central zone of each rhizobox. To maintain soil moisture, all the 30 rhizoboxes were watered every day using equal amounts of tap water, which was the same as the actual irrigation water. Weeds were manually removed from rhizoboxes. The rhizospheric soils and bulk soils in each rhizobox were collected using 1-ml plastic syringes without tips. And each soil sample was transferred into a 2-ml centrifuge tube, which was placed on ice. All the soil samples were immediately stored at − 80 °C before DNA extraction. Soil samples were collected every other week from the first to the ninth week. The five sampling stages correspond to cotyledon (S1), 1st node (S2), 3rd node (S3), nth node (S4), and blooming stage (S5). All the soil samples were immediately stored at − 80 °C before DNA extraction. In total, 300 soil samples were collected (five fertilization treatments × two compartments × five stages × six replicates).

### DNA extraction, sequencing, and processing

About 0.5 g soil from each sample was used for the extraction of genomic DNA using the FastDNA SPIN Kit for Soil (MP Biomedicals, Santa Ana, CA). The V3-V4 region of the bacterial 16S rRNA gene was targeted for amplification with primers 338F and 806R (Liu et al. 2016a). Amplification and product purification were preformed according to a previous study (Mei et al. 2019). The PCR products were quantified using a Qubit Fluorometer (Invitrogen, Carlsbad, CA, USA), and were equally mixed to construct libraries. The quantity of amplicon libraries was assessed using the Library Quantification Kit for Illumina (Kapa Biosciences, Woburn, MA, USA). High-throughput sequencing of amplicon libraries was performed on the Illumina MiSeq platform (Illumina Inc., San Diego, USA) at the LC-Bio Technology Co., Ltd. (Hangzhou, Zhejiang, China).

Paired-end reads were truncated by cutting off the sample-unique barcode and primer sequences, and then merged using FLASH. Quality filtering was performed according to the default settings in fqtrim v.0.94 (Bokulich et al. 2013). Vsearch v.2.13.4 was used to remove chimeric sequences (Rognes et al. 2016). The sequence counts obtained from each sample ranged from 21,572 to 69,396 with a median of 37,770. Then the high-quality sequences with ≥97% similarity were assigned to 9452 operational taxonomic units (OTUs) using Vsearch. The taxonomic classification of the representative sequences of OTUs was determined based on the Ribosomal Database Project (RDP) classifier (Maidak et al. 2000). OTUs assigned to chloroplasts were discarded to avoid the debatable classification of cyanobacterial taxa.

To detect the shifts in bacterial community abundance, we filtered OTUs to reduce noise as previously described (Evans and Wallenstein 2014). Those OTUs present in over half of the replicates (e.g., four of six) were deemed ‘present’, while those OTUs absent in over half of the replicates were deemed ‘absent’. Undefined OTUs present in three replicates were excluded together with consistently absent OTUs among six replicates under one fertilization treatment in one compartment. The species that responded consistently to one treatment in six replicates were retained to explore the assembly dynamics of bacterial communities over time. We further searched for the temporally specifically-selected OTUs from these filtered OTUs. The OTUs with the highest and lowest relative abundances at one specific stage were identified as the specifically enriched and inhibited OTUs at this stage, respectively (Wu et al. 2019). In this study, the temporally specifically-selected OTUs were identified from each compartment under each nitrogen treatment. The difference in the number of the specifically-selected OTUs at each stage was analyzed using independent t test.

### Quantitative PCR (qPCR) of nitrogen functional genes

To analyze variations in the abundance of nitrogen functional genes in response to nitrogen management and soybean development, we performed qPCR with three replicates on a QuantStudio™ 6 Flex Real-Time PCR System (Life Technologies Corporation, Carlsbad, CA, USA) using the SYBR Green II method. The mixture of genomic DNA obtained from every six replicates was used as the qPCR template. The nitrogen-cycling processes and functional genes analyzed in this study included nitrogen fixation (*nifH*), nitrification (*AOB amoA*, *nxrA*, and *comammox clade A*), denitrification (*napA*, *nirK*, *qnorB*, and *nosZ*), anaerobic ammonium oxidation (anammox; *hzsB* and 16S rRNA gene of anammox bacteria), and dissimilatory nitrate reduction to ammonium (DNRA; *nrfA*). The abundance of total bacteria was assessed by the copy numbers of 16S rRNA gene. The qPCR experiment was carried out according to the previous research (Shu et al. 2019). The qPCR primer sequences and reaction conditions were summarized in Supplementary Table S1.

### Data analyses

All the software packages used in this study were conducted in R environment v.4.0.0 (https://www.r-project.org), and the visualization of graphics were accomplished with the *ggplot2* package (http://ggplot2.tidyverse.org) unless otherwise indicated. The dissimilarities of bacterial communities and nitrogen functional genes between different treatment groups were tested via the analysis of similarities (ANOSIM) and permutational multivariate analysis of variance (PERMANOVA). The principle coordinate analysis (PCoA) of community structure was based on Bray-Curtis distance metrics. These analyses were performed using the *vegan* package (https://github.com/vegandevs/vegan).

The gene abundance differences in different groups were tested using kruskal.test with adjusted *P* value by the Benjamini and Hochberg method. The log-transformed copy number of each functional gene was visualized using the *pheatmap* package (https://CRAN.R-project.org/package=pheatmap). *pheatmap* package was also used to present the temporal shifts in microbial community abundance under different compartments and fertilization treatments.

To explore the ecological interactions of temporally specifically-selected OTUs, we calculated species-species Spearman’s rank coefficients (*ρ*) using the *Hmisc* package (https://hbiostat.org/R/Hmisc), and the adjusted *P* value (*q*) was calculated based on the false discovery rate method using the *fdrtool* package (http://strimmerlab.org/software/fdrtool). Significantly correlated OTUs (*q* < 0.05, *ρ* > 0.7) were used to construct the networks of specifically selected sub-communities based on the correlations of OTU abundance variations on time scales. The topological properties (average clustering coefficient, average path length, and modularity) of observed and random networks were calculated using the *igraph* package (Csardi and Nepusz 2006).

We further identified keystone OTUs according to their among- and within-module connectivity using the criteria (c-score > 0.6 and/or z-score > 2.5) reported in previous studies (Agler et al. 2016; Guimera and Amaral 2005). The sub-networks of keystone taxa were visualized with Gephi (Bastian et al. 2009). To verify whether keystone taxa dominate the interactions among the temporally specifically-selected OTUs, we calculated the proportions of inter- and intra-phylum negative correlations between the phyla containing keystone taxa in the whole networks. Furthermore, we analyzed the correlations of the average relative abundances of key phyla at the overall community level. The goodness-of-fit and statistical significance of linear regression model were measured using the *plyr* package (Wickham 2011). The relationships between the relative abundances of key phyla and nitrogen-cycling genes were analyzed using redundancy analysis with the *vegan* package. The copy numbers of genes were transformed to their natural logarithms after adding one.

To explore the linkages between taxonomic and functional changes, we estimated the relative importance of temporally specifically-selected OTUs to nitrogen functional genes using the *randomForest* package (Liaw and Wiener 2002). The predictive power of the random forest model was statistically tested using the *rfUtilities* package (Murphy et al. 2010). The OTUs that significantly predicted functional genes were defined as functional groups. The percentage of the functional groups being specifically enriched or inhibited were shown in pie charts using the *corrplot* package (https://github.com/taiyun/corrplot).

## Supplementary Information

**Additional file 1.**

## Data Availability

The dataset analyzed during the current study is available at GenBank’s Sequence Read Archive under Bioproject accession number PRJNA675200.
