# Supplementary material for: Inter-phylum negative interactions affect soil bacterial community dynamics and functions during soybean development under long-term nitrogen fertilization
Source: Stress Biol. 2021 Nov 26;1(1):15. doi: 10.1007/s44154-021-00015-0 (PMC10441860; doi:10.1007/s44154-021-00015-0)
Supplement: Supplementary file 1 — Additional file 1. [file 44154_2021_15_MOESM1_ESM.docx]

**Supplementary Information**

**Inter-phylum negative interactions affect soil bacterial community dynamics and functions during soybean development under long-term nitrogen fertilization**

*Submitted to* ***Stress Biology***

Chunfang Zhang ^1^, Shuo Jiao ^1^, Duntao Shu ^1,*^, Gehong Wei ^1,*^

^1^State Key Laboratory of Crop Stress Biology for Arid Areas, Shaanxi Key Laboratory of Agricultural and Environmental Microbiology, College of Life Sciences, Northwest A&F University, Yangling, Shaanxi 712100, China

^*^Correspondence: [donald.shu@nwafu.edu.cn](file:///D:\1greenhouse-1\Stress%20biology\donald.shu@nwafu.edu.cn) (DT Shu); <weigehong@nwsuaf.edu.cn> (GH Wei)

**Contents**

**1. Figures**

**Figure S1** Diagram of the rhizobox (not to scale)

**Figure S2** Principal coordinate analysis (PCoA) of bacterial communities between different compartments and treatments on time scales. Coefficient of determination (*R*^2^) is calculated using permutational multivariate analysis of variance (PERMANOVA). ^***^*P* <0.001; B, bulk soil; R, rhizosphere; CK, no fertilizer; N1, low urea input; N2, high urea input; O1, low sheep manure input; O2, high sheep manure input

**Figure S3** Pairwise associations between key phyla based on their relative abundances in (**a**) rhizosphere and (**b**) bulk soil at the community level. Trend lines and goodness-of-fit indexes are added when relationships between variables are significant in linear models. ^*^*P* <0.05; ^**^*P* <0.01; ^***^*P* <0.001; CK, no fertilizer; N1+N2, urea input; O1+O2, sheep manure input

**Figure S4** Redundancy analysis based on the correlations between key phyla and nitrogen functional genes at the community level. CK, no fertilizer; N1, low urea input; N2, high urea input; O1, low sheep manure input; O2, high sheep manure input; *TB_16S*, 16S rRNA genes of total bacteria; *Amx_16S*, 16S rRNA genes of anammox bacteria; *AOB*, *AOB amoA*; *comamoA*, *comammox clade A*

**Figure S5** Linear regression relationships between gene copies and IncMSE (increase in mean squared error) values. Trend line and goodness-of-fit index (*R*^2^) are added. ***P <0.001; *P <0.05

**Figure S6** Box plots of (**a**) gene abundances and (**b**) their IncMSE values. The same means separation letters in a single figure indicate that the values are not significantly different at *P* <0.05. IncMSE, increase in mean squared error; CK, no fertilizer; N1, low urea input; N2, high urea input; O1, low sheep manure input; O2, high sheep manure input

**2. Tables**

**Table S1** Primers and reaction conditions used for quantitative PCR

**Table S2** Permutational multivariate analysis of variance (PERMANOVA) of bacterial community structures

**Table S3** Significance test of kmeans clusterings for the analyses in Fig. 1a

**Table S4** Independent t test for the difference in the number of the specifically-selected OTUs

**Table S5** Key network features of temporally specifically-selected sub-communities

**Table S6** Differences of soil properties under different nitrogen treatments

**Figure S1**

**
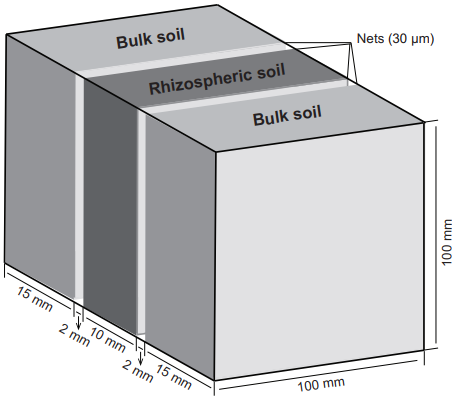
**

**Figure S1** Diagram of the rhizobox (not to scale)

**Figure S2**

**
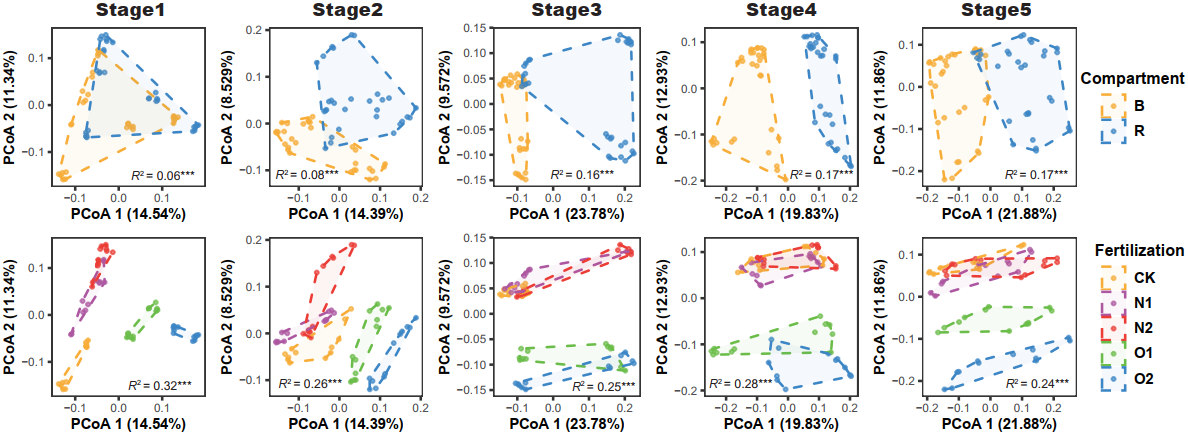
**

**Figure S2** Principal coordinate analysis (PCoA) of bacterial communities between different compartments and treatments on time scales. Coefficient of determination (*R*^2^) is calculated using permutational multivariate analysis of variance (PERMANOVA). ^***^*P* <0.001; B, bulk soil; R, rhizosphere; CK, no fertilizer; N1, low urea input; N2, high urea input; O1, low sheep manure input; O2, high sheep manure input

**Figure S3**

**
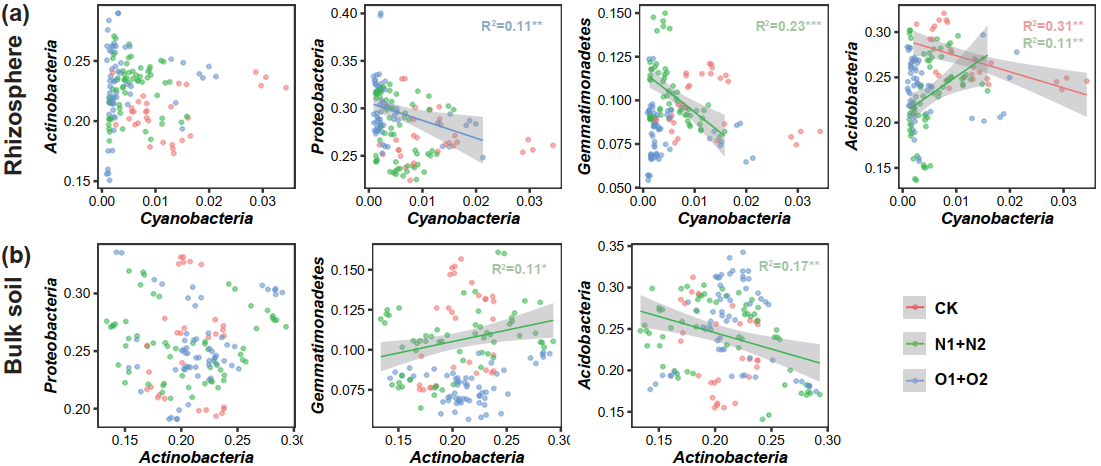
**

**Figure S3** Pairwise associations between key phyla based on their relative abundances in (**a**) rhizosphere and (**b**) bulk soil at the community level. Trend lines and goodness-of-fit indexes are added when relationships between variables are significant in linear models. ^*^*P* <0.05; ^**^*P* <0.01; ^***^*P* <0.001; CK, no fertilizer; N1+N2, urea input; O1+O2, sheep manure input

**Figure S4**


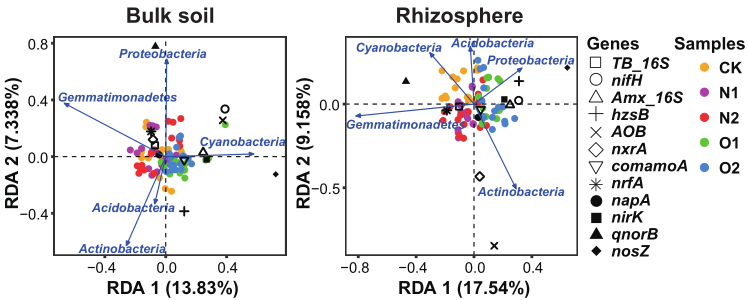


**Figure S4** Redundancy analysis based on the correlations between key phyla and nitrogen functional genes at the community level. CK, no fertilizer; N1, low urea input; N2, high urea input; O1, low sheep manure input; O2, high sheep manure input; *TB_16S*, 16S rRNA genes of total bacteria; *Amx_16S*, 16S rRNA genes of anammox bacteria; *AOB*, *AOB amoA*; *comamoA*, *comammox clade A*

**Figure S5**


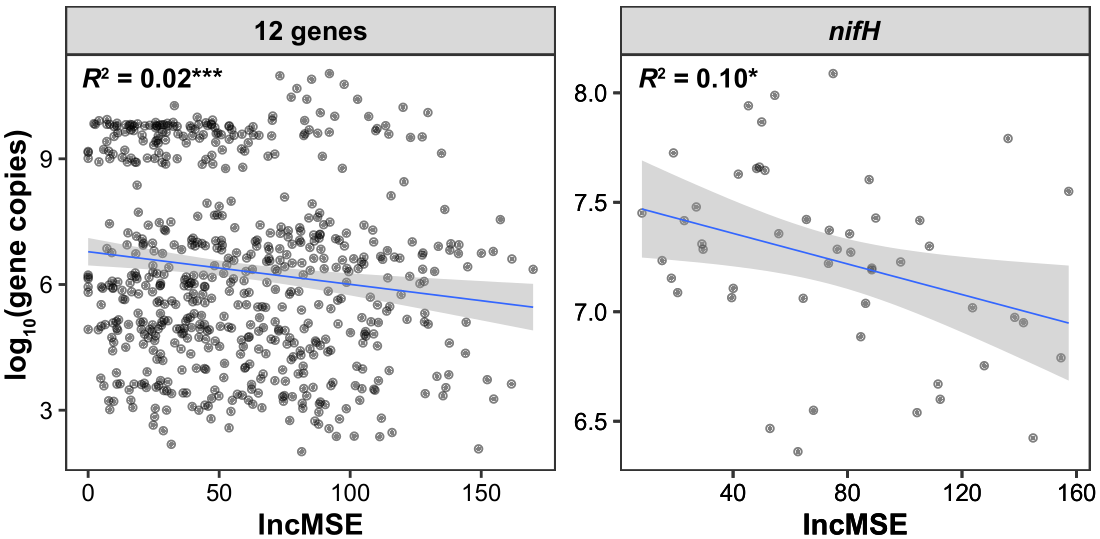


**Figure S5** Linear regression relationships between gene copies and IncMSE (increase in mean squared error) values. Trend line and goodness-of-fit index (*R*^2^) are added. ***P <0.001; *P <0.05

**Figure S6**


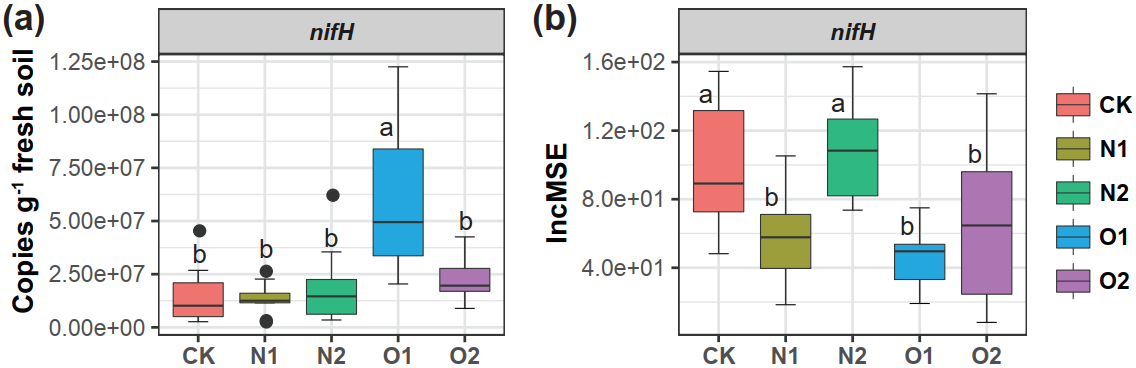


**Figure S6** Box plots of (**a**) gene abundances and (**b**) their IncMSE values. The same means separation letters in a single figure indicate that the values are not significantly different at *P* <0.05. IncMSE, increase in mean squared error; CK, no fertilizer; N1, low urea input; N2, high urea input; O1, low sheep manure input; O2, high sheep manure input

**Table S1** Primers and reaction conditions used for quantitative PCR

| **Target gene** | **Sequence of primer pair (5’-3’)** | **Annealing (°C)** | **Thermal cycling program** |
| --- | --- | --- | --- |
| Total bacteria | 341F: CCTACGGGAGGCAGCAG | 60 | 5 min at 95°C, followed by 35 cycles of 30 s at 95°C, 30 s at 60°C, and 40 s at 72°C |
|  | 518R: ATTACCGCGGCTGCTGG |  |  |
| *AOB amoA* | amoAF: GGGGTTTCTACTGGTGGT | 55 | 3 min at 94°C, followed by 35 cycles of 30 s at 94°C, 30 s at 55°C, and 45 s at 72°C |
|  | amoAR: CCCCTCKGSAAAGCCTTCTTC |  |  |
| *qnorB* | qnorB2F :GGNCAYCARGGNTAYGA | 56 | 10 min at 95°C, followed by 35 cycles of 15 s at 95°C, 60 s at 56°C, and 30 s at 72°C |
|  | qnorB5R: ACCCANAGRTGNACNACCCACCA |  |  |
| *nirK* | NirK583F: TCATGGTGCTGCCGCGKGACGG | 64 | 15 min at 95°C, followed by 35 cycles of 15 s at 95°C, 60 s at 64°C,and 30 s at 72°C |
|  | NirK909R: GAACTTGCCGGTKGCCCAGAC |  |  |
| *Anammox* | Amx809f: GCCGTAAACGATGGGCACT | 60 | 10min at 95°C, followed by 35 cycles of 60 s at 95°C, 60 s at 60°C, and 45 s at 72°C |
|  | Amx1066r: AACGTCTCACGACACGAGCTG |  |  |
| *napA* | napA3F: CCCAATGCTCGCCACTG | 60 | 5 min at 95°C, followed by 35 cycles of 60 s at 95°C, 60 s at 60°C, and 45 s at 72°C |
|  | napA3R: CATGTTKGAGCCCCACAG |  |  |
| *nrfA* | nrfA2F: CACGACAGCAAGACTGCCG | 60 | 5 min at 95°C, followed by 35 cycles of 60 s at 95°C, 60 s at 60°C, and 45 s at 72°C |
|  | nrfa2R: CCGGCACTTTCGAGCCC |  |  |
| *nxrA* | F1norA: CAGACCGACGTGTGCGAAAG | 57 | 10 min at 95°C, followed by 35 cycles of 15 s at 95°C, 30 s at 57°C, and 30 s at 72°C |
|  | R1norA: TCYACAAGGAACGGAAGGTC |  |  |
| *hzsB* | HZSBeta396F: ARGGHTGGGGHAGYTGGAAG | 59 | 10 min at 95°C, followed by 35 cycles of 15 s at 95°C, 60 s at 59°C, and 30 s at 72°C |
|  | HZSBeta742R: GTYCCHACRTCATGVGTCTG |  |  |
| *nosZ* | nosZ1F: WCSYTGTTCMTCGACAGCCAG | 60 | 10 min at 95°C, followed by 35 cycles of 15 s at 95°C, 60 s at 60°C, and 30 s at 72°C |
|  | nosZ1R: ATGTCGATCARCTGVKCRTTYTC |  |  |
| *nifH* | nifH-F: AAAGGYGGWATCGGYAARTCCACCAC | 53 | 10 min at 95°C, followed by 35 cycles of 15 s at 95°C, 60 s at 53°C, and 30 s at 72°C |
|  | nifH-R: TTGTTSGCSGCRTACATSGCCATCAT |  |  |
| comamoA | comamoA-F: AGGNGAYTGGGAYTTCTGG | 58 | 3 min at 95°C, followed by 35 cycles of 15 s at 94°C, 30 s at 58°C, and 30 s at 72°C |
|  | comamoA-R:CCGVACATACATRAAGCCCAT |  |  |

**Table S2** Permutational multivariate analysis of variance (PERMANOVA) of bacterial community structures

| **Group** | **Stage** | ***R*^2^** | ***P*** |
| --- | --- | --- | --- |
| CK+N1+N2 | S1 | 0.2434 | <0.001 |
|  | S2 | 0.2009 | <0.001 |
|  | S3 | 0.2026 | <0.001 |
|  | S4 | 0.2052 | <0.001 |
|  | S5 | 0.1734 | <0.001 |
| O1+O2 | S1 | 0.1657 | <0.001 |
|  | S2 | 0.1060 | 0.002 |
|  | S3 | 0.1069 | 0.006 |
|  | S4 | 0.1365 | 0.011 |
|  | S5 | 0.1178 | 0.005 |

^a^Abbreviations: CK, no fertilizer; N1, low urea input; N2, high urea input; O1, low sheep manure input; O2, high sheep manure input. S1, S2, S3, S4, and S5 represent one, three, five, seven, and nine weeks post transplantation, respectively

**Table S3** Significance test of kmeans clusterings for the analyses in Fig. 1a

| **Compartment** | **Treatment** | **kmeans=3** | |  | **kmeans=4** | |
| --- | --- | --- | --- | --- | --- | --- |
|  |  | ***R*** | ***P*** |  | ***R*** | ***P*** |
| Bulk soil | CK | 0.7683 | <0.001 |  | 0.5517 | <0.001 |
|  | N1 | 0.7607 | <0.001 |  | 0.5076 | <0.001 |
|  | N2 | 0.5628 | <0.001 |  | 0.5602 | <0.001 |
|  | O1 | 0.7662 | <0.001 |  | 0.5750 | <0.001 |
|  | O2 | 0.6759 | <0.001 |  | 0.4525 | <0.001 |
| Rhizosphere | CK | 0.5809 | <0.001 |  | 0.4192 | <0.001 |
|  | N1 | 0.4962 | <0.001 |  | 0.4505 | <0.001 |
|  | N2 | 0.4998 | <0.001 |  | 0.4495 | <0.001 |
|  | O1 | 0.4832 | <0.001 |  | 0.4688 | <0.001 |
|  | O2 | 0.5192 | <0.001 |  | 0.4160 | <0.001 |

^a^Abbreviations: CK, no fertilizer; N1, low urea input; N2, high urea input; O1, low sheep manure input; O2, high sheep manure input

**Table S4** Independent t test for the difference in the number of the specifically-selected OTUs

| **Compartment** | **Stage** | **Enrichment  (Mean±SE)** | **Inhibition  (Mean±SE)** | ***P*** |
| --- | --- | --- | --- | --- |
| Bulk soil | S1 | 96±10 | 33±6 | <0.001 |
|  | S2 | 159±10 | 66±8 | <0.001 |
|  | S3 | 167±22 | 123±15 | 0.143 |
|  | S4 | 85±8 | 150±18 | 0.010 |
|  | S5 | 33±6 | 96±10 | <0.001 |
| Rhizosphere | S1 | 69±11 | 56±3 | 0.312 |
|  | S2 | 143±10 | 161±24 | 0.516 |
|  | S3 | 168±24 | 156±30 | 0.750 |
|  | S4 | 114±16 | 74±9 | 0.064 |
|  | S5 | 56±3 | 69±11 | 0.312 |

^a^Abbreviations: S1, S2, S3, S4, and S5 represent one, three, five, seven, and nine weeks post transplantation, respectively

**Table S5** Key network features of temporally specifically-selected sub-communities

| Compartment | Treatment | Edge | Vertex | Empirical | | |  | Random | | |
| --- | --- | --- | --- | --- | --- | --- | --- | --- | --- | --- |
|  |  |  |  | ACC | APL | Md |  | ACC | APL | Md |
| Bulk soil | CK | 1816 | 452 | 0.36 | 3.77 | 0.53 |  | 0.02 | 3.16 | 0.28 |
|  | N1 | 1549 | 358 | 0.38 | 3.40 | 0.35 |  | 0.03 | 2.96 | 0.26 |
|  | N2 | 2106 | 378 | 0.46 | 3.74 | 0.33 |  | 0.03 | 2.71 | 0.24 |
|  | O1 | 2643 | 438 | 0.43 | 3.58 | 0.43 |  | 0.03 | 2.70 | 0.22 |
|  | O2 | 1740 | 365 | 0.50 | 3.91 | 0.30 |  | 0.03 | 2.85 | 0.25 |
| Rhizosphere | CK | 1045 | 365 | 0.32 | 4.15 | 0.52 |  | 0.01 | 3.57 | 0.34 |
|  | N1 | 829 | 223 | 0.46 | 3.33 | 0.40 |  | 0.04 | 2.92 | 0.31 |
|  | N2 | 2474 | 495 | 0.50 | 4.19 | 0.45 |  | 0.02 | 2.94 | 0.25 |
|  | O1 | 2587 | 501 | 0.37 | 3.81 | 0.43 |  | 0.02 | 2.90 | 0.24 |
|  | O2 | 2673 | 459 | 0.38 | 3.59 | 0.32 |  | 0.02 | 2.75 | 0.22 |

^a^Abbreviations: CK, no fertilizer; N1, low urea input; N2, high urea input; O1, low sheep manure input; O2, high sheep manure input; ACC, average clustering coefficient; APL, average path length; Md, modularity

**Table S6** Differences of soil properties under different nitrogen treatments

| Soil property | CK | N1 | N2 | O1 | O2 | *P* |
| --- | --- | --- | --- | --- | --- | --- |
| pH | 8.64 | 8.59 | 8.53 | 8.55 | 8.48 | 0.010 |
| total carbon (g kg^-1^) | 17.08 | 17.14 | 17.50 | 20.71 | 21.76 | 0.012 |
| total nitrogen (g kg^-1^) | 1.16 | 1.28 | 1.32 | 1.52 | 1.64 | 0.009 |
| soil organic matter (g kg^-1^) | 19.02 | 21.49 | 21.18 | 27.36 | 27.59 | 0.014 |
| nitrate nitrogen (mg kg^-1^) | 2.39 | 2.70 | 3.92 | 2.59 | 2.98 | 0.016 |
| ammonium nitrogen (mg kg^-1^) | 5.47 | 4.47 | 5.00 | 7.71 | 5.49 | 0.012 |
| available phosphorus (mg kg^-1^) | 28.77 | 19.93 | 22.13 | 34.43 | 34.80 | 0.010 |
| available potassium (mg kg^-1^) | 154.30 | 156.40 | 170.80 | 196.53 | 265.60 | 0.011 |
| available iron (mg kg^-1^) | 9.57 | 10.33 | 10.23 | 12.29 | 12.37 | 0.014 |

^a^Abbreviations: CK, no fertilizer; N1, low urea input; N2, high urea input; O1, low sheep manure input; O2, high sheep manure input
